# Properties of Local Interactions and Their Potential Value in Complementing Genome-Wide Association Studies

**DOI:** 10.1371/journal.pone.0071203

**Published:** 2013-08-05

**Authors:** Wenhua Wei, Attila Gyenesei, Colin A. M. Semple, Chris S. Haley

**Affiliations:** 1 MRC Human Genetics Unit, MRC Institute of Genetics and Molecular Medicine at the University of Edinburgh, Edinburgh, United Kingdom; 2 Finnish Microarray and Sequencing Centre, Turku Centre for Biotechnology, University of Turku and Åbo Akademi University, Turku, Finland; University of Southern California, United States of America

## Abstract

Local interactions between neighbouring SNPs are hypothesized to be able to capture variants missing from genome-wide association studies (GWAS) via haplotype effects but have not been thoroughly explored. We have used a new high-throughput analysis tool to probe this underexplored area through full pair-wise genome scans and conventional GWAS in diastolic and systolic blood pressure and six metabolic traits in the Northern Finland Birth Cohort 1966 (NFBC1966) and the Atherosclerosis Risk in Communities study cohort (ARIC). Genome-wide significant interactions were detected in ARIC for systolic blood pressure between *PLEKHA7* (a known GWAS locus for blood pressure) and *GPR180* (which plays a role in vascular remodelling), and also for triglycerides as local interactions within the 11q23.3 region (replicated significantly in NFBC1966), which notably harbours several loci (*BUD13*, *ZNF259* and *APOA5*) contributing to triglyceride levels. Tests of the local interactions within the 11q23.3 region conditional on the top GWAS signal suggested the presence of two independent functional variants, each with supportive evidence for their roles in gene regulation. Local interactions captured 9 additional GWAS loci identified in this study (3 significantly replicated) and 73 from previous GWAS (24 in the eight traits and 49 in related traits). We conclude that the detection of local interactions requires adequate SNP coverage of the genome and that such interactions are only likely to be detectable between SNPs in low linkage disequilibrium. Analysing local interactions is a potentially valuable complement to GWAS and can provide new insights into the biology underlying variation in complex traits.

## Introduction

The study of gene-gene interactions (epistasis) in complex traits has seen rapid advances in recent years. The potential importance of epistasis in explaining the extent and basis of heritability has been emphasized in both model organisms [1], [2] and humans [3]. Previously searching for epistasis in genome-wide association studies (GWAS) was limited by the substantial demands it placed upon computational resources. The development of new methods and tools has greatly reduced the computational barrier and made the routine analysis of epistasis in GWAS data achievable [4], [5], [6], [7], [8]. Furthermore, progress has been made in dissecting the molecular mechanisms underlying epistasis [9], [10]. With these advances it is hoped that future studies will accumulate more evidence of epistasis and improve our understanding of the role of epistasis in the genetic regulation of complex traits [11].

New developments such as BiForce support high-throughput analysis of epistasis in GWAS data allowing full pair-wise interactions for multiple traits in multiple populations to be quickly computed [4], [12]. The new challenge is to identify reliable epistatic signals with plausible functional mechanisms from the high throughput interaction results. Several issues can complicate this challenge. First, previous studies suggest that most GWAS populations may have relatively low power for the detection of epistasis in complex traits [3], [12], [13], [14], i.e. one may in general have to work with sub-significant interaction results. Second, detection and subsequent replication of a pair-wise interaction requires SNPs to be in strong linkage disequilibrium (LD) with the causal variants at each locus in both discovery and replication samples, making replication more difficult than in the case of a single association signal from GWAS [15], [16], [17]. A high density of SNPs genotyped would help by providing a good LD coverage but many GWAS populations were actually genotyped with lower density SNP chips (e.g. <400 000 SNPs). Third, a big proportion of epistatic SNPs (e.g. >40%) may not be near a gene [15], [16] so bioinformatics methods considering non-coding variants are needed to assess their functional roles [18], [19].

Various approaches may be considered to increase detection power for epistasis. One reason for the low power issue is the use of stringent genome-wide significance thresholds derived from Bonferroni adjustment for often several billions of pair-wise tests of all SNP combinations. Several knowledge-driven methods select a subset of SNPs based on prior biological knowledge (e.g. genes and proteins in particular pathways) and only test pair-wise interactions between the selected SNPs so that a more relaxed threshold could be used to claim significance [6], [20], [21]. Knowledge-driven methods that are restricted to SNPs with functional annotation will miss interaction signals involving other SNPs, such as those in pathways not currently implicated, or those lacking functional annotation altogether (e.g. SNPs in non-coding regions). Interactions between neighbouring SNPs (local interactions) are hypothesized to be able to capture variants missing from GWAS via haplotype effects [22]. Local interactions have been previously reported (without testing for replication) in several human diseases and metabolic traits [12], including C-reactive protein (CRP), diastolic blood pressure (DBP), glucose (GLU), high-density lipoprotein (HDL), insulin (INS), low-density lipoprotein (LDL), systolic blood protein (SBP), triglycerides (TRI), but they are not thoroughly explored. Concentrating only on local interactions between SNPs on the same chromosome and within a certain distance such as one million base pairs (Mb) would also mean a much reduced number of pair-wise tests and consequently a relaxed significance threshold. On the other hand, it has been shown that analysing multiple metabolic traits together could identify pleiotropic effects and common pathways from the shared single SNP signals (not necessarily genome-wide significant) from GWAS [23], [24]. It is an open question whether sub-significant epistatic signals shared in multiple metabolic traits could also lead to new insights into the functional organization of the complex metabolomes [25].

Here we used the Atherosclerosis Risk in Communities study cohort (ARIC) and the Northern Finland Birth Cohort (NFBC1966) to explore the potential values of high throughput analyses of epistasis in the eight metabolic traits above. ARIC is one of the largest GWAS populations available and both its sample size and density of SNPs genotyped nearly double the counterparts in NFBC1966. After data scrutiny and quality control checks (Table S1), we performed full pair-wise genome scans using BiForce and conventional GWAS in all eight metabolic traits in both cohorts, identified and tested replication of genome-wide significant epistatic signals. It has been shown that a combined search algorithm implemented in BiForce can increase the power of detection of epistasis by applying appropriate thresholds to test interactions involving SNPs with genome-wide significant marginal effects (marginal SNPs) while keeping false-positive rates under control [12]. We then assessed the impact of sample size and SNP density on power of detection by comparing the computed interaction profiles in each trait between the two cohorts. Further we characterised local interactions between SNPs located within 1 Mb and with an interaction P value (P_int_) less than a threshold of 1.0E-05 derived from region based permutations (Material and Methods section). We used the r^2^ measure of LD throughout this study which is considered to be the best LD measure in studying epistasis and robust to the Hardy-Weinberg Equilibrium assumption [26]. Our results suggest that analysing local interactions is an effective and valuable complement to GWAS and can provide new insights into the biology underlying variation in complex traits.

## Results

### Pair-wise genome scans detect significant epistasis

We analyzed 514 662 and 323 697 SNPs in the ARIC and NFBC1966 cohorts respectively (Table S1). For single SNP based genome scans (i.e. conventional GWAS) the consensus threshold (P = 5.0E-08) [27] was applied to identify marginal SNP. For full pair-wise genome scans Bonferroni adjusted thresholds for the total number of tests, i.e. 3.8E-13 and 9.5E-13 when no marginal SNPs were involved and 9.7E-08 and 1.5E-07 when at least one marginal SNP was involved, were used to identify genome-wide significant epistatic SNP pairs in ARIC and NFBC1966 respectively (Materials and Methods section). Conventional GWAS identified numerous genome-wide significant SNPs in five traits (i.e. CRP, GLU, HDL, LDL and TRI) in both cohorts (Table S2). These results are in line with the original GWAS of the two cohorts [28], [29], [30].

Pair-wise genome scans identified six epistatic pairs of SNPs carrying strong interaction signals in ARIC only (Table 1). The first two pairs, i.e. rs409354 - rs1417733 for SBP and rs10892020 - rs17119975 for TRI, had mainly interactions with negligible marginal effects and were considered genome-wide significant based on the Bonferroni adjusted threshold of 3.8E-13 (note the P_int_ of the SBP pair did not exceed but was close enough to the stringent threshold). The remaining four epistatic pairs each included one marginal SNP (Table S2), of which the two SNP pairs with P_int_<1.9E-09 for TRI were genome-wide significant and the remaining two (for TRI and HDL respectively) were suggestive. Interestingly, the four epistatic SNP pairs identified for TRI were all local interactions between SNPs closely located (distance <45 kilobases) in the 11q23.3 region, which contains multiple genes associated with lipid traits (Table 1). The SNP pair rs409354 - rs1417733 for SBP was also found in DBP but with P_int_ of 3.9E-06. Replication of the six epistatic pairs was tested in the NFBC1966 cohort but only at the region level (Materials and Methods section) because none of the listed epistatic SNPs were genotyped in NFBC1966. All six pairs had some evidence for replication (P_int_<0.05) but only the replication of the 11q23.3 local interaction pairs for TRI exceeded the significance threshold of 5.6E-04 derived from permutation.

**Table 1:** Genome-wide significant epistatic pairs identified in the ARIC cohort and their replication in the NFBC199 cohort.*

Trait	SNP_1_	chr_1_	pos_1_	gene_1_	SNP_2_	chr_2_	pos_2_	gene_2_	P_int_	rep_SNP_1_	rep_SNP_2_	rep_ P_int_
SBP	rs409354	11	16 876 618	PLEKHA7	rs1417733	13	95 327 273	near GPR180	4.3E-13	rs10832696	rs942149	7.2E-03
TRI	rs10892020	11	116 589 652	near *BUD13*	rs17119975	11	116 634 557	*BUD13*	6.5E-16	rs7123583	rs2075295	2.1E-04
TRI	rs3741298^a^	11	116 657 561	*ZNF259*	rs7396835	11	116 684 028	near *APOA4*	1.0E-09	rs7123583	rs2075295	2.1E-04
TRI	rs3741298^a^	11	116 657 561	*ZNF259*	rs7396851	11	116 684 164	near *APOA4*	1.8E-09	rs7123583	rs2075295	2.1E-04
TRI	rs12799766^a^	11	116 558 427	near *BUD13*	rs10892020	11	116 589 652	near *BUD13*	6.0E-09^b^	rs7123583	rs2075295	2.1E-04
HDL	rs1285884	6	7 143 075	*RREB1*	rs247617^a^	16	56 990 716	near *CETP*	2.8E-08^b^	rs11755724	rs7499892	3.5E-02

* : genome-wide significant thresholds for interactions involving marginal SNPs were 2.1E-09 for TRI and 3.5E-09 for HDL; SNP_1_ (SNP_2_), chr_1_ (chr_2_), pos_1_ (pos_2_), gene_1_ (gene_2_) – name, chromosome, position and mapped gene of the first (second) SNP; P_int_ – P value of the interaction test; rep_SNP_1_ (rep_SNP_2_, rep_P_int_) – the first (second, interaction P value) SNP of the best replicated pair;

a : the genome-wide significant single SNP with marginal effects;

b : genome-wide suggestive.

### SNP coverage is critical in the detection of local interactions

**Figure 1:**
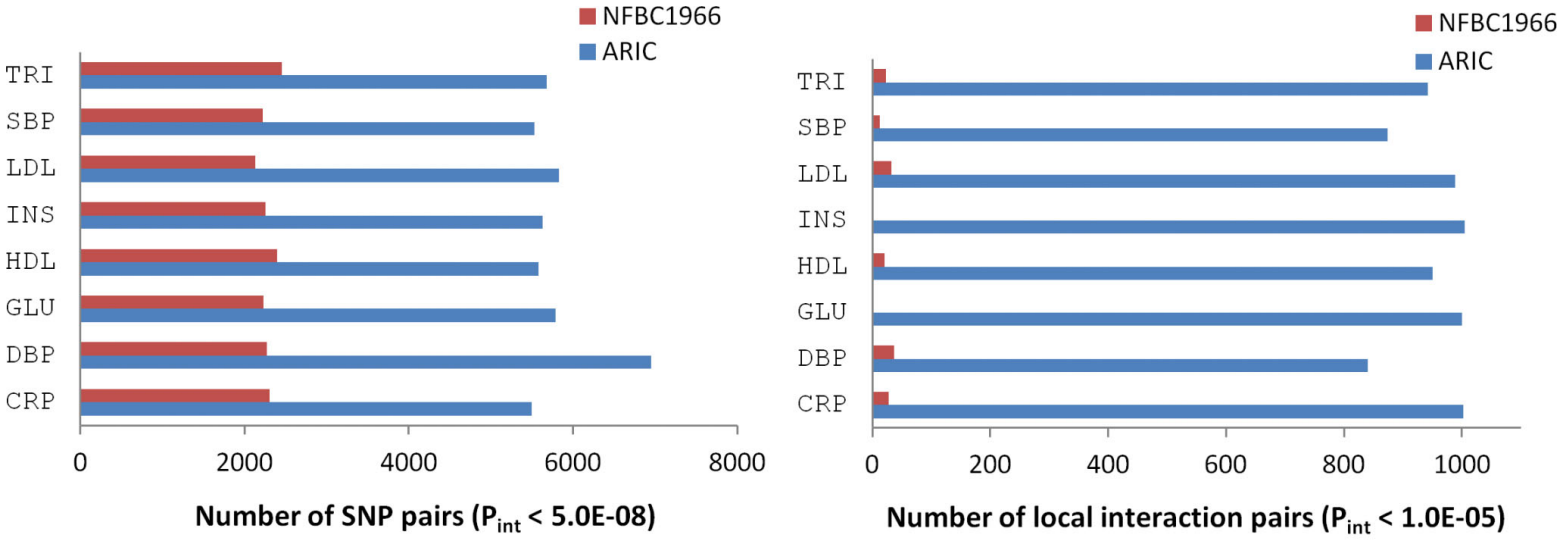
Differences in the numbers of SNP pairs with P_int_<5.0E-08 and local interaction pairs (P_int_<1.0E-05) detected in each trait between ARIC and NFBC1966.

The power advantage in ARIC over NFBC1966 was clearly observed in every trait studied when sub-significant SNP pairs were considered together (Figure 1, Table S3). For example, the numbers of SNP pairs with P_int_<5.0E-08 (i.e. the GWAS consensus threshold) in each trait in NFBC1966 were approximately 40% of the counterparts in ARIC, which is coincident with the difference of the SNP coverage in the two cohorts, i.e. (323 697 in NFBC1966)^2^/(514 662 in ARIC)^2^ = 0.4. The most striking differences were the numbers of local interaction pairs detected in each trait between NFBC1966 (<50) and ARIC (800 to 1000), suggesting that SNP coverage might be particularly important to detect local interactions.

Using the TRI trait in ARIC as an example, we examined the impact of sample size and SNP coverage separately. A reduction of the ARIC sample size by random sampling (with no changes to SNP coverage) to 4873 – the sample size of NFBC1966 (Table S1) - did not substantially alter the number of SNP pairs with P_int_<5.0E-08 (from 5684 to 5446) or the number of local interaction pairs (from 942 to 960), but did reduce the interaction signals of the top four pairs for TRI listed in Table 1 dramatically (P_int_ values reduced to 6.1E-07, 5.2E-06, 5.3E-06 and 1.7E-04 respectively, none remaining significant). However, a reduction by random sampling of the number of ARIC SNPs (with no changes to sample size) to 323 697 – the total number of SNPs in NFBC1966 – dramatically reduced the numbers of SNP pairs with P_int_<5.0E-08 (to 2376) and local interaction pairs (to 332), including all the four SNP pairs for TRI (Table 1). Clearly, SNP coverage is disproportionately important for the power of epistasis detection.

### Local interactions capture both known and novel loci via haplotype effects

**Figure 2:**
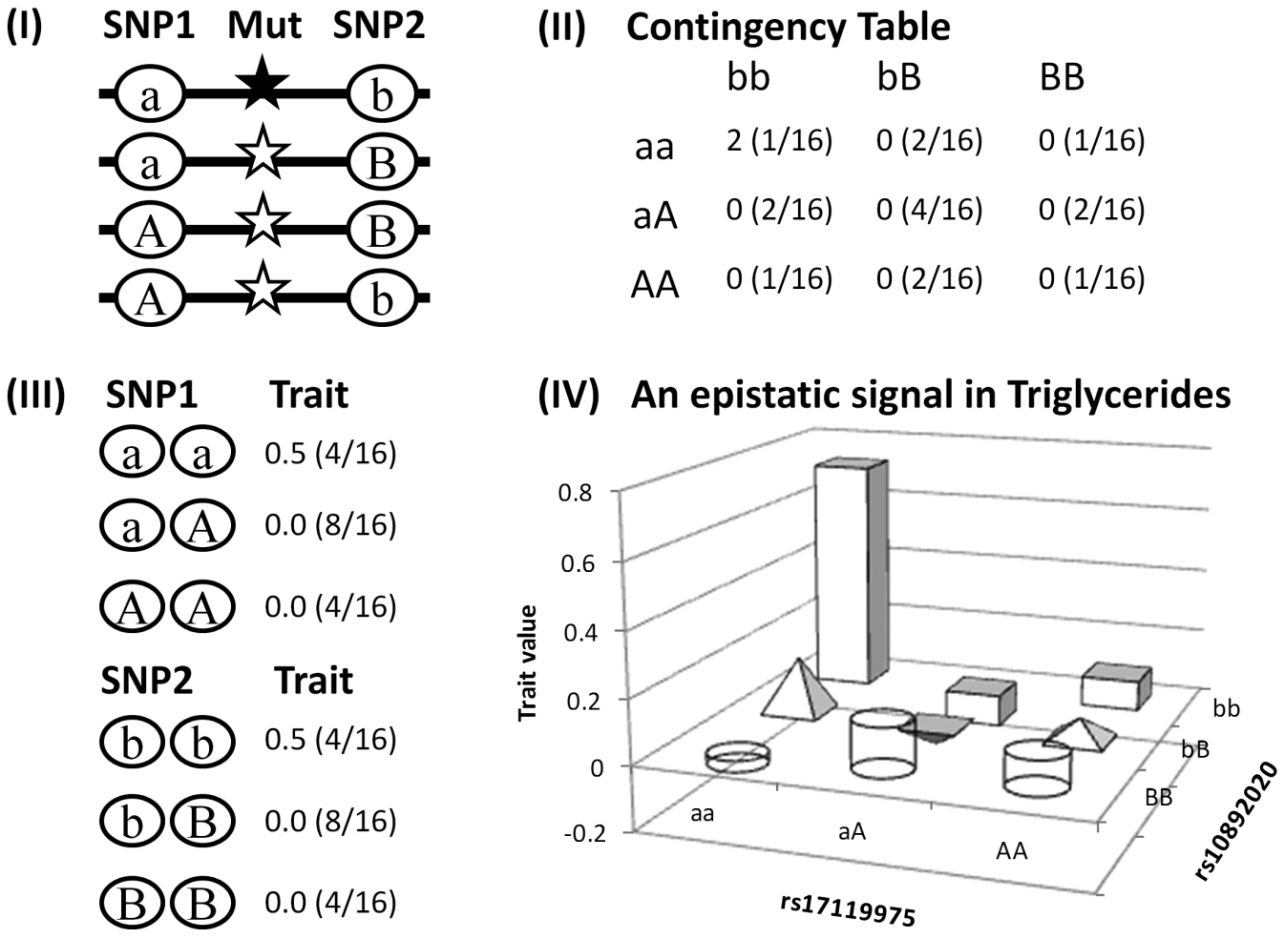
A cartoon model illustrating a haplotype tagging a recessive causal variant can generate an apparent statistical interaction between two unlinked SNPs each with limited marginal effects. (I) A recessive causative variant (black star) is associated with only the ***ab*** SNP haplotype, assuming Hardy-Weinberg Equilibrium, i.e. an equal allele frequency of 0.5 for each SNP so there is no LD between the two SNPs and an equal frequency of 0.25 for each of the four possible haplotypes, and the causal variant with an effect size of 1. (II) Only individuals homozygous for this haplotype (***ab***/***ab***) are genetically differentiated generating apparent epistasis (averaged trait value and joint genotype frequency in the bracket in each cell). (III) Marginal effects associated with the individual SNPs are limited with only one in four individuals of the ***aa*** or ***bb*** SNP genotype being affected with a trait value of 2 so the averaged trait value of the genotype is 0.5 (SNP genotype frequency in brackets), thus the individual SNPs may not be detected by a conventional GWAS. (IV) This resembles the interaction between rs17119975 and rs10892020 in TRI (Table 1) where neither SNP had important marginal effects and their interaction signal was mainly because of the differentiated phenotype associated with the double homozygous ***aabb*** genotype.

Exploring the pairwise interactions with P_int_<1.0E-05 but which do not reach genome-wide significance it is clear that local interactions comprised only a small proportion (<0.1%) of the total number of SNP pairs retained per trait in both cohorts (Table S3). However local interactions covered various regions across the genome and could be useful in identifying important loci including those missing from GWAS. To illustrate this point, we created a cartoon model describing a haplotype tagging a recessive causal variant can generate an apparent statistical interaction between two unlinked SNPs each with limited marginal effects under the assumption of Hardy-Weinberg Equilibrium (i.e. equal allele frequency of 0.5 for each SNP and equal haplotype frequency of 0.25 for the four possible haplotypes) (Figure 2). Under this model, only individuals with the ***aabb*** genotype (i.e. homozygous for the ***ab*** haplotype carrying the causal variant) show differentiated phenotypes which leads to an apparent statistical interaction signal in a contingency table based test whereas conventional GWAS can not detect the causal variant from the associations with either SNP. The model resembled the interaction between rs17119975 and rs10892020 in TRI (Table 1) where both SNPs had limited marginal effects and their interaction signal mainly came from the double homozygous genotype (Figure 2). Several GWAS significant SNPs were identified between the epistatic pairs of SNPs (Table S2), indicating that local interactions can capture important marginal effects.

**Figure 3:**
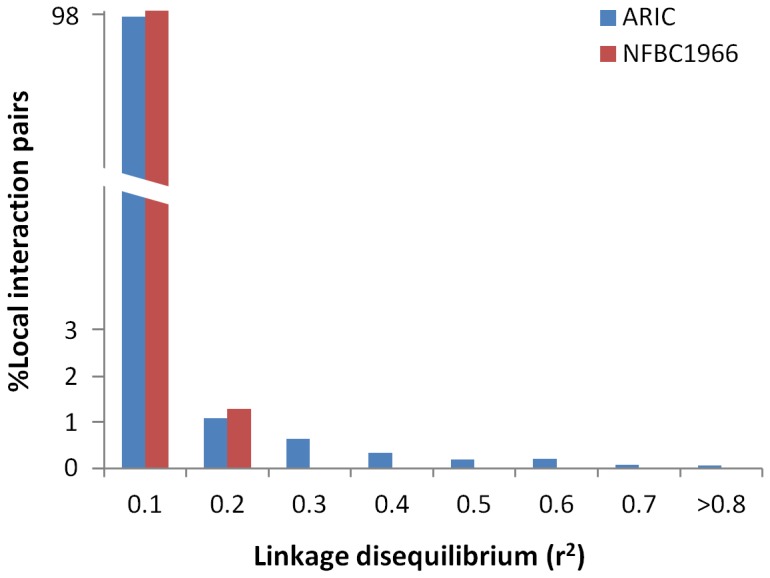
Proportions of local interactions in different LD ranges.

We computed LD for all the local interaction pairs in the two cohorts and plotted a histogram of the proportions of local interaction pairs in different LD bins (Figure 3). Clearly, the vast majority of local interactions had a low LD (r^2^<0.2) and only a few had r^2^>0.5 each with a generally moderate interaction signal (i.e. P_int_<1.0E-06). Local interactions were distributed rather evenly across the ranges of distances between two epistatic SNPs and most P_int_ values in ARIC were less than 1.0E-06 with only 0.2% with P_int_<1.0E-08 (Figure S1).

**Figure 4:**
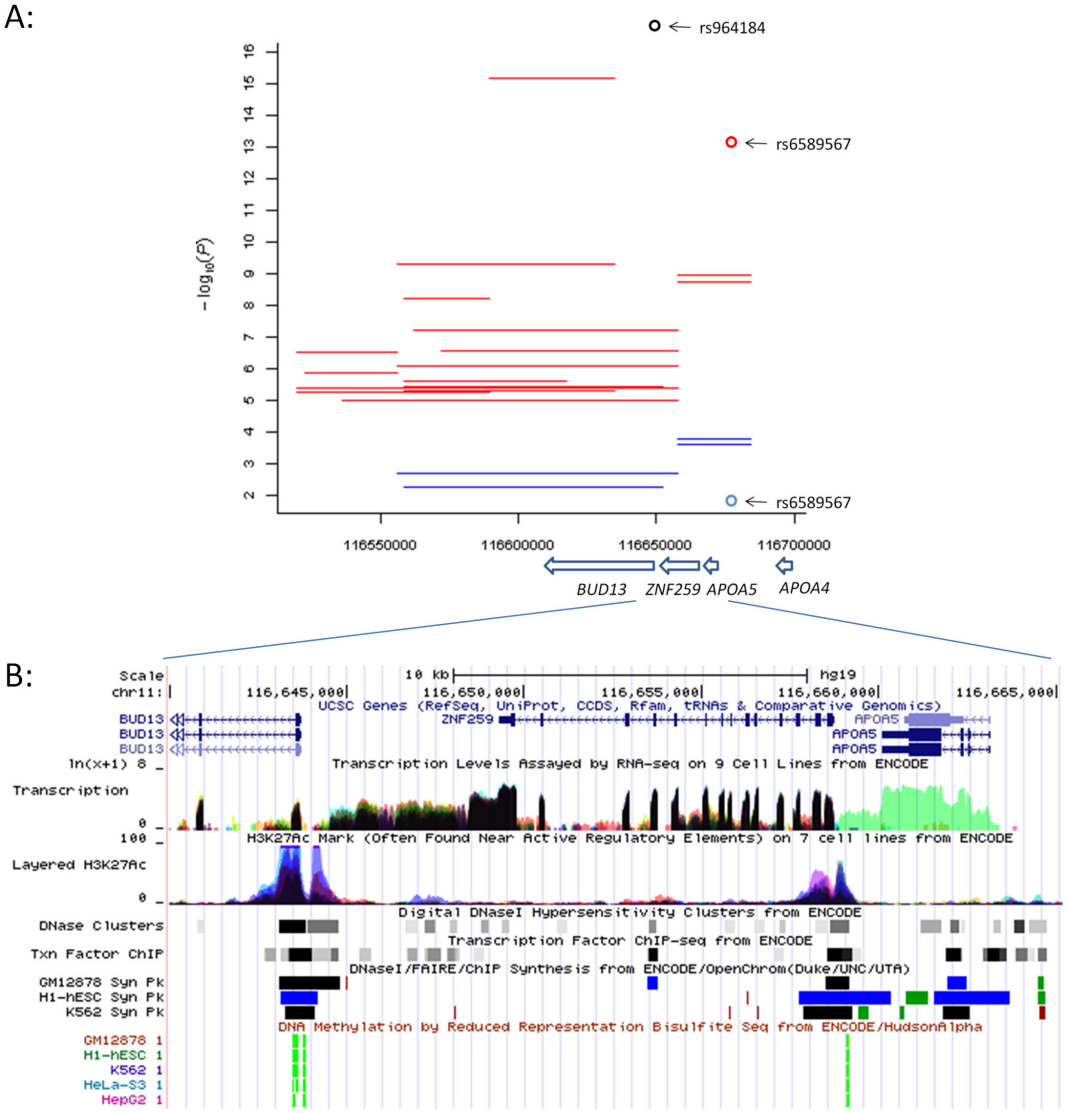
Local interactions (P_int_<1.0E-05) within the 11q23.3 region associated with TRI in ARIC and supporting ENCODE regulatory evidence. (A) black oval: the top marginal SNP rs964184 within the region; each line representing an interaction between two SNPs at the start and end locations where red and blue lines represent interactions prior to and post conditional tests respectively; red and blue ovals: the marginal SNP rs6589567 prior to and post conditional test respectively; y axis: association P values in the −log_10_ scale; x axis: genomic location in base pair; arrow bar showing transcription direction and location of the gene (italic) below the bar. (B) a snapshot from UCSC genome browser showing clustered ENCODE regulatory elements aligning to the 5′ ends of *BUD13* and *ZNF259* respectively.

The LD (r^2^) values of the four local interaction pairs for TRI in ARIC (Table 1) were also in the low range: rs17119975 – rs10892020 (0.44), rs3741298 – rs7396835 (0.05), rs3741298 – rs7396851 (0.05) and rs12799766 – rs10892020 (0.40). We then aligned all local interactions within the 11q23.3 region associated with TRI in ARIC (including the four Table 1 pairs) and found they did not always overlap with each other (Figure 4A). Conditional tests of each of these local interactions by fitting the top marginal SNP rs964184 in the region (P = 2.5E-38, Table S2) as the background found the interactions in rs17119975 – rs10892020 and rs12799766 – rs10892020 disappeared (P_int_>0.05, thus the interactions were explained by the marginal SNP) but the interactions in four SNP pairs remained significant (P_int_<1.0E-02, thus the interactions were statistically independent to the marginal SNP): rs3741298 – rs7396835 (or rs7396851) covering *ZNF259* and *APOA5*, rs17092638 – rs3741298 and rs12799766 – rs4417316 covering *BUD13* and *ZNF259* (Figure 4A). The same conditional tests of each of the remaining 13 marginal SNPs within the region for TRI (Table S2) found only rs6589567 with a P value (2.9E-02) less than 0.05, which located near *APOA5* and between the epistatic SNPs of the first two independent pairs (Figure 4A). Further conditional tests of each of the first two independent pairs by fitting rs6589567 as the background showed their interactions were also independent (P_int_ of 1.5E-07 and 2.4E-07 respectively) to that SNP. In addition, we found two clusters of ENCODE regulatory elements [31] aligning to the 5′ ends of *BUD13* and *ZNF259* (Figure 4B) and captured by the independent pairs respectively. These results are consistent with the possibility that local interactions might tag at least two independent functional variants in the region.

The ANNOVAR [19] region-based annotation found that 63% of the local interaction SNP pairs in ARIC (61% in NFBC1966) mapped loci reported in previously published GWAS. These included nine loci that were genome-wide significant in the GWAS analyses in this study (Table 2, Table S2), of which only *CETP* for HDL and *BUD13*–*ZNF259*–*APOA5* for TRI (Table 1) were the top GWAS loci in the individual traits studied, suggesting not all top GWAS loci (i.e. with the strongest marginal effects) were involved in local interactions. Interestingly, *CETP* was captured by local interactions in both cohorts; *LPL* and *BUD13*–*ZNF259*–*APOA5* were captured in both HDL and TRI (Table 1) in ARIC. Most of these local interactions in Table 2 had some evidence of replication in the counterpart cohort (P_int_<0.05) of which the three pairs tagging *LDLR* and *TOMM40* – *APOE* for LDL in ARIC and *CETP* for HDL in NFBC1966 respectively were significantly replicated.

**Table 2:** Local interactions captured additional genome-wide significant loci identified in GWAS of the eight traits in ARIC and/or NFBC1966.*

Region	gene	Trait	SNP_1_	pos_1_	SNP_2_	pos_2_	P_int_	LD (r^2^)	rep_SNP_1_	rep_SNP_2_	rep_ P_int_
2p24.1	*APOB*	LDL	rs427021	21451458	rs386397	21451827	9.5E-10	0.26	rs10206521	rs312046	1.9E-03
5q13.3	*HMGCR*	LDL	rs2006760	74562029	rs1559203	75449814	7.0E-06	0	rs6866661	rs10072459	1.1E-02
8p21.3	*LPL*	HDL	rs1441766	19862788	rs7461115	19871540	9.7E-07	0.22	rs894210	rs2410630	1.1E-02
8p21.3	*LPL*	TRI^b^	rs1441766	19862788	rs7013777	19878356	1.2E-06	0.23	rs10099160	rs10103634	1.0E-03
11q21-q14.3	*MTNR1B*	GLU	rs10765558	92493781	rs56247942	92999977	5.7E-06	0	rs505423	rs1374475	3.3E-03
11q23.3	*ZNF259,APOA5*	HDL	rs3741298	116657561	rs7396835	116684028	1.8E-07	0.05	Nil	Nil	Nil
12q24.31	near *HNF1A* a	CRP	rs2708104	121483949	rs1718161	121627458	1.5E-06	0.00	rs11065408	rs2230912	3.4E-02
16q13	*CETP*	HDL	rs9989419^c^	56985139	rs12708980	57012379	5.6E-06	0.00	rs7499892	rs4784744	1.2E-02
16q13-q21	*CETP* a	HDL	rs9989419^c^	56985139	rs4783999	57651985	6.7E-06	0.03	rs2518054	rs12708990	4.5E-04
19p13.2	*LDLR*	LDL	rs12052058^c^	11159525	rs1799898	11227554	1.8E-06	0.10	rs11668477	rs2228671	2.6E-04
19q13.32	*TOMM40, APOE*	LDL	rs4803750^c^	45247627	rs4803759	45327459	4.4E-06	0.02	rs4803750	rs4803760	3.4E-04

* : SNP_1_ (SNP_2_), pos_1_ (pos_2_) – name and position the first (second) SNP; P_int_ – P value of the interaction test; rep_SNP_1_ (rep_SNP_2_, rep_P_int_) – the first (second, interaction P value) SNP of the best replicated pair; LD: r^2^ linkage disequilibrium between two epistatic SNPs.

a : detected in NFBC1966 and test replication in ARIC;

b : region shared in multiple traits;

c : genome-wide significant marginal SNP.

Local interactions also pointed to GWAS loci that were missed in our GWAS analyses, including 24 each associated with one of the eight traits studied here and 49 each associated with a related trait (Table S4). These 73 loci, particularly the 49 could be considered as novel loci for our GWAS analyses of the eight traits. For example, in the 2q31.1-q24.3 region marked by local interactions in TRI, previous GWAS identified *G6PC2*, *ABCB11* and *LRP2* associated with various metabolic traits and other biochemical traits [32], [33], [34], [35]; *ERAP1* and a haplotype of *ERAP1* and *ERAP2* (5q15) marked by local interactions in CRP were reported to be associated with Ankylosing Spondylitis where CRP levels are considered as one of the clinical indicators of inflammatory activities of patients [36], [37], [38]; the *CPS1* gene (2q34) marked by local interactions in DBP was previously found responsible for susceptibility to persistent pulmonary hypertension function [39], [40]. Again, we found a number of local interaction regions showing pleiotropic effects in correlated traits, e.g. *KLKB1* (4q35.2) and *ARL15* (5q11.2) in HDL and TRI; *CD34* (1q32.2) and *MYO16* (13q33.3) in DBP and SBP; *VPS13C* – *C2CD4B* (15q22.2), *ZFAND6* (15q25.1) and *WWOX* (16q23.2-q23.1) in GLU and INS; *SYCP2L* – *ELOVL2* (6p24.2-p24.1) in LDL and CRP; *CCDC92* – *ZNF664* (12q24.31) in LDL, CRP and DBP. In addition, we found the *PCSK9* - *USP24* (1p32.3-p32.2) region was marked by local interactions in LDL in both the ARIC and NFBC1966 cohorts.

## Discussion

Compared to the great success in GWAS, high throughput analysis of epistasis is both in its infancy and substantially more challenging in detection as well as interpretation. Indeed, as shown in this study, conventional GWAS identified genome-wide significant SNPs in multiple loci in five out of the eight traits studied in both cohorts that were relatively easy to replicate (Table S2), whereas significant epistasis signals were detected only in TRI and SBP in ARIC – one of the biggest GWAS cohorts and moderately replicated (significantly for the TRI signals) in NFBC1966. However, high throughput pair-wise genome scans enabled us to investigate the value of local interactions in identifying potentially important loci from sub-significant epistatic results. We showed that local interactions could capture loci with important marginal effects (e.g. via haplotypes) and were useful to better understand the genetic structures underlying such loci (i.e. the 11q23.3 region) as well as to identify 73 loci missing from the accompanying GWAS. Furthermore, it was possible to generate promising hypotheses about the regulatory mechanisms underlying independent statistical signals of epistasis, via interrogation of ENCODE and other genomic sequence annotations. Our results suggest that studying epistasis is a potentially valuable complement to GWAS and can provide new insights into the biology of complex traits, particularly those (i.e. DBP, SBP and INS) where no significant signals were detected in the accompanying GWAS.

Low power of detection is the key issue in studying epistasis in single GWAS populations. We showed that in addition to sample size, SNP coverage was critical to power as it generates the detectable levels of LD required for epistasis detection and replication. The NFBC1966 cohort used a smaller sample size and a lower SNP coverage and this likely contributed to the many fewer epistatic SNP pairs than in ARIC based on the same criteria. Furthermore, in testing replication of the significant signals (Table 1) in NFBC1966 SNP proxies of the epistatic SNPs had to be used because the epistatic SNPs were not genotyped in that cohort, which likely reduced the chance of replication. Imputation could help improve statistical replication but in this case it would be recommended to accommodate population specific LD patterns owing to for example isolation in NFBC1966. Other factors (e.g. population structure, allele frequency variation and environmental factors) are also known to influence power [15]. For example, individuals in the NFBC1966 cohort were much younger (31 years old) than those in the ARIC cohort (45 to 64 years old) and thus had quite different metabolic profiles for the traits studied, which might have posed additional difficulty in detection and replication. Therefore, it would be worthwhile to further test replication of at least the significant signals in other cohorts with a good SNP coverage because they are pleiotropic and biologically meaningful. For example, the interaction between *PLEKHA7* and *GPR180* for SBP (and DBP) may suggest an interesting model of blood pressure regulation, where *PLEKHA7* is a GWAS locus associated with blood pressure [41] and *GPR180* is a G protein-coupled receptor produced predominantly in vascular smooth muscle cells and may play an important role in the regulation of vascular remodelling [42] (Table 1). It has been shown that *PLEKHA7* codes adherens junction proteins binding paracingulin regulating RhoA and Rac1 activities [43] which may involve various G protein-coupled receptors including *GPR180*.

Using a high density SNP chip for GWAS genotyping would mean even more stringent genome-wide significance thresholds based on Bonferroni adjustment and thus a further reduction of the power of detection of epistasis. Such Bonferroni adjusted thresholds can hardly remain practical as many more SNPs derived from the increasingly popular sequencing studies are used as input to future GWAS. There is a clear need for the community to define consensus genome-wide significance thresholds for future epistasis studies. A recent effort based on Illumina's HumanHap 550 bead SNP chip and Monte Carlo simulations has made a good progress towards this goal and suggests that an adjustment of 44% of the total number of pair-wise tests is appropriate to avoid using an overly stringent threshold [44]. To fully achieve the goal further work is needed to examine the impact of SNP density and other factors (e.g. sample size) on the correlation structure underlying billions pair-wise tests in studying epistasis in GWAS.

Our local interaction results provide fresh evidence supporting the hypothesis that some genetic variations in complex traits may be captured by epistasis between neighbouring SNPs [22] and shed light on a new search path for variants missing from GWAS based on a more relaxed threshold than the genome-wide thresholds derived by Bonferroni adjustment. We showed clearly that local interactions were not driven by high LD between a pair of SNPs (Figure 3) but more likely by haplotypes of SNPs in low LD or unlinked as we previously predicted [12]. Local interaction pairs reserve the usual interpretation of haplotypes, i.e. physical coupling of alleles on the DNA strand inherited from a single parent [26], but the alleles are unlinked or weakly linked and thus may be more powerful than single SNPs particularly when the genotyped SNPs are not in high LD with a causal variant tagged by haplotypes (Figure 2).

Previously we argued that it was unlikely to be able to distinguish a marginal signal captured by a haplotype from a genuine local interaction using statistical approaches alone [12], i.e. fitting the marginal signal could largely diminish the local interaction signal. In the 11q23.3 region example (Figure 4), fitting the top marginal SNP did remove the signal of the top local interaction pair but not the signals of the four independent pairs coinciding with ENCODE evidence of regulatory elements aligning to the 5′ ends of *BUD13* and *ZNF259* respectively. From a statistical viewpoint one may conclude that the top local interaction pair probably captured a marginal signal without interaction but the independent pairs could be real interactions. However, the mechanisms underlying these local interactions could be complicated. For example, the marginal signal captured by the top pair, if true, may also remove the interaction signals of two independent pairs overlapped the region marked by the top pair (Figure 4). Follow-up functional studies are needed to find out whether these local interactions are real or simply capture functional variants without interactions. In addition, our results indicating pleiotropic epistatic signals suggest that analysing multiple related traits together may be a useful approach to uncover functionally important loci, which also requires further investigation in the future.

## Materials and Methods

### Study cohorts and ethics statement

This study was approved by the institutional review board of the West of Scotland Research Ethics Service of NHS in the UK. The GWAS data of both the NFBC1966 and ARIC studies were provided by the NIH Database of Genotype and Phenotype via specific Data Use Certifications issued by the Data Access Committee of the National Heart, Lung and Blood Institute. Both studies have been described in detail elsewhere [29], [30]. Briefly, the NFBC1966 study cohort recruited subjects born in two Northern Finland provinces (i.e. Oulu and Lapland) in 1966 and was approved by the Ethical Committee of the Northern Ostrobothnia Hospital District and all participants gave written informed consent. At the age of 31 each subject provided fasting blood samples for evaluation of the metabolic measures and was genotyped with Illumina Infinium 370cnvDuo array and assessed for blood pressure and other traits [30]. The ARIC study cohort recruited adults aged 45 to 64 years from four US communities in 1987–89 each was genotyped with Genome-Wide Human SNP Array 6.0 and underwent baseline examination and fasting blood sample tests and follow-up examinations and tests in approximately every three years in four field centres. The ARIC study was approved by the institutional review board of each field centre institute and all participants gave written informed consent in accordance with the Declaration of Helsinki [29], where only subjects of European descent were considered in this study.

In both cohorts, a standard procedure was used to measure height, weight, sitting SBP and DBP for each participant; lipid traits (i.e. HDL, LDL and TRI) were measured using standard enzymatic methods [29], [30]. In NFBC1966, CRP, GLU and INS were analyzed using immunoenzymometric assay (Medix Biochemica), a glucose dehydrogenase method (Granutest 250, Diagnostica Merck) and radioimmunoassay (Pharmacia Diagnostics) respectively [30]. In ARIC, serum CRP, GLU and INS were assessed using the immunoturbidimetric CRP-Latex (II) high-sensitivity assay from Denka Seiken (Tokyo, Japan) [28], a hexokinase/glucose-6-phosphate dehydrogenase method on a Coulter DACOS device (Beckman Coulter, Fullerton, CA) and radioimmunoassay (125Insulin kit; Cambridge Medical Diagnosis, Bilerica, MA) [45], respectively.

Subjects were excluded from the analysis of each individual trait if matching the phenotypic exclusion criteria defined in the original GWAS of NFBC1966 [30]: had missing values of phenotypes or covariates detailed below (all traits); used diabetic medication or gave blood samples without fasting (GLU, HDL, INS, LDL, TRI); were diabetic or pregnant or phenotypic values were in excess of three standard deviations from the mean (GLU, INS). Most of the traits in ARIC used in this study were measured at the first visit except that CRP was measured at the fourth visit because the sample sizes in previous visits were fairly small. Relevant covariates for CRP were all based on the fourth visit.

A common protocol was used to perform quality control over both cohorts using the GenABEL package [46] implemented in R (http://www.r-project.org/): individual call rate at 97%, SNP call rate at 95%, minor allele frequency at 2%, P value for deviation from Hardy-Weinberg equilibrium at 1.0E-10, false discovery rate for unacceptably high individual heterozygosity at 0.01. In addition, to control population stratification, individuals were excluded if they were outliers of one of the first three principal components (false discovery rate of 0.005) calculated (using the R function *cmdscale*) from the identity-by-state matrix constructed using the GenABEL *ibs* function. We analysed SNPs on the autosomal chromosomes only. After quality control, 514 662 and 323 697 SNPs were left in the ARIC and NFBC1966 cohorts respectively with various numbers of individuals in different traits (Table S1). For each cohort/trait, the identity-by-state matrix was then reconstructed and the first ten principal components were calculated and stored for statistical analyses.

### Statistical analysis

In NFBC1966 each trait was adjusted for gender, oral contraception and pregnancy. In ARIC, each trait was adjusted for gender, age, oral contraception and field centre; the three lipid traits (i.e. HDL, LDL and TRI) were also adjusted for two additional effects: taking of cholesterol-lowering medication within two weeks of the visit and taking of medications that secondarily affect cholesterol. After adjustment for covariates, each trait was normalised using the GenABEL *rntransform* function and then adjusted for relatedness and the first ten principal components using the GenABEL *polygenic* function, and the resultant environmental residuals (i.e. *pgresidualY*) were used as the actual trait values to test for association [47]. The polygenic heritability was also calculated for each trait at this stage and these are shown in Table S1.

Conventional GWAS analyses (i.e. assuming additive effects only) of each trait in each cohort were conducted using the GenABEL *mmscore* function and the consensus threshold (P = 5.0E-08) [27] was applied to declare a SNP with genome-wide significant marginal effects. The inflation factors (computed by regression of observed association P values against the expected) in each genome scan were all between 1 and 1.03, suggesting relatedness among individuals and potential population stratification in each cohort were well accounted for. BiForce was used to perform full pair-wise genome scans for each trait in each cohort and retained SNP pairs with an interaction P value (P_int_) less than 1.0E-05. Bonferroni adjusted thresholds as previously defined [12] were used to identify genome-wide significant epistatic SNP pairs. Given *N* to be the total number of SNPs with *K* (*K*>0) marginal SNPs detected in the conventional GWAS, the 5% genome-wide thresholds were derived as P = 0.05/(*N*×(*N*–1)/2-(*N*–1)×*K*)) for a full pair-wise genome scan (i.e. 9.5E-13 and 3.8E-13 in NFBC1966 and ARIC respectively) and P = 0.05/((*N*–1)×*K*) for interactions involving at least one marginal SNP (i.e. 1.5E-07 and 9.7E-08 if *K* is 1 in NFBC1966 and ARIC respectively).

Identified epistatic SNP pairs were tested for replication in independent samples at the SNP and/or region levels following our previous protocol [15] to accommodate the issues of different SNP coverage and LD patterns across study cohorts. The SNP level replication is possible only if both SNPs of an epistatic pair were genotyped in the independent samples and considered significant if the P value of the interaction between the two SNPs exceeded the 5% nominal threshold in independent samples. The region level replication tested interactions between each of ten adjacent SNPs (i.e., five upstream and five downstream) of the two SNPs involved in the epistatic interaction and used the 5% thresholds derived from permutation (i.e. permute the phenotypes and test all pair-wise interactions within the region 1000 times) to declare significance of the best replicate SNP pair, i.e. the pair of SNPs with the lowest P_int_ value. If either SNP of an epistatic pair was not genotyped in the independent samples, the nearest SNP was chosen as its proxy to perform the region level replication tests for the pair.

The detection of local interactions may be substantially affected by LD patterns varying across the genome and thus require a different threshold to declare significance. We used permutation of the TRI trait in the ARIC cohort as an example to investigate such a threshold based on a window of 41 SNPs on the same chromosome (i.e. 20 upstream and 20 downstream of a SNP randomly sampled from the genome), which may not necessarily mark a region in exactly one million base pairs but capture the LD pattern within the region and with a fixed number of tests. For each randomly sampled SNP, we iteratively permuted the phenotypes and tested interactions of every pair-wise combination of SNPs within the window 100 times and recorded the lowest P_int_ value in each iteration to derive the 5% P_int_ value. We randomly sampled 200 SNPs from the genome and calculated the average of the derived 5% P_int_ values as the threshold as 1.3E-04, which was indeed less stringent than 6.1E-05 based on the Bonferroni adjustment (i.e. 0.05/(41*40/2)) or 1.0E-05 used to retain epistatic SNP pairs during the BiForce scans. The permutation derived threshold was based on one region at a time that was not adjusted for the total number of local interaction regions in the genome which is unknown in advance. For simplicity, we used 1.0E-05 as the threshold to declare local interactions in this study.

All SNP positions were based on the current human genome build (UCSC hg19/NCBI 37.3). Local interactions were extracted from the retained epistatic pairs if both SNPs located on the same chromosome and within a distance less than 1 Mb (i.e. P_int_<1.0E-05) and their LD values were calculated. The functional annotation tool ANNOVAR [19] was used to map local interactions to loci reported in GWAS Catalog [48], where we identified each GWAS locus located within a genomic region bounded by the two SNPs of a local interaction SNP pair. ANNOVAR was also used to map SNPs to genes using a window of 20 kilobases upstream and 20 kilobases downstream of the SNP. The RegulomeDB [18], [49] and UCSC Genome Browser (http://genome.ucsc.edu/) were used to search for regulatory elements published by the ENCODE project [31].

## Supporting Information

Figure S1 Distributions of local interactions in different ranges of interaction P values or distances between a pair of SNPs.
(TIF)

Table S1 Summary of the eight metabolic traits in ARIC and NFBC1966.
(XLSX)

Table S2 Genome-wide significant single SNPs in ARIC and NFBC1966.
(XLSX)

Table S3 Profiling epistatic SNP pairs detected in eight metabolic traits in ARIC and NFBC1966.
(XLSX)

Table S4 Local interactions tag GWAS loci identified from external studies of the same or highly related traits.
(XLSX)
