# Supplementary figures and images for: Properties of Local Interactions and Their Potential Value in Complementing Genome-Wide Association Studies
Source: PLoS One. 2013 Aug 5;8(8):e71203. doi: 10.1371/journal.pone.0071203 (PMC3733963; doi:10.1371/journal.pone.0071203)

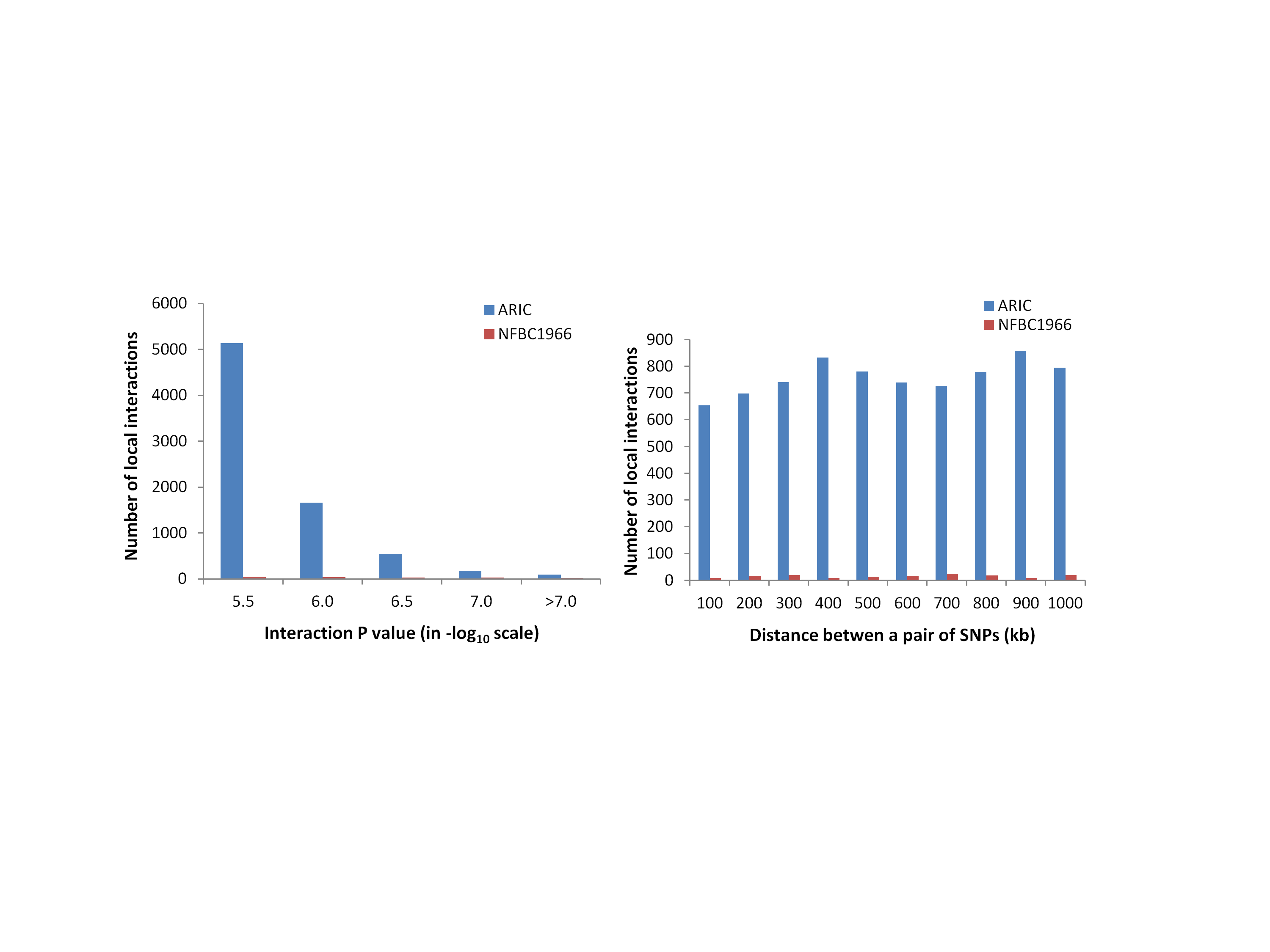

Supplement: Figure S1 — Distributions of local interactions in different ranges of interaction P values or distances between a pair of SNPs. (TIF) [file pone.0071203.s001.tif]
